# Lipopolysaccharide exposure induces oxidative damage in *Caenorhabditis elegans*: protective effects of carnosine

**DOI:** 10.1186/s40360-020-00455-w

**Published:** 2020-12-03

**Authors:** Jing Ma, Xiaoyuan Xu, Ranran Wang, Haijing Yan, Huijuan Yao, Hongmei Zhang, Shaowei Jiang, Ajing Xu

**Affiliations:** 1 Department of Pharmacy, Xinhua Hospital, Shanghai Jiaotong University School of Medicine, Shanghai, 200092 P. R. China; 2 Department of Endocrinology, Xinhua Hospital, Shanghai Jiaotong University School of Medicine, Shanghai, 200092 P. R. China; 3 Institute for metabolic and neuropsychiatric Disorders, Binzhou Medical University Hospital, Binzhou, China; 4 Emergency Department, Xinhua Hospital, Shanghai Jiaotong University School of Medicine, Shanghai, 200092 P. R. China

**Keywords:** Carnosine, *Caenorhabditis elegans*, Lipopolysaccharide, Oxidative stress, p38 MAPK

## Abstract

**Background:**

The present study was designed to investigate the protective effects and mechanisms of carnosine on lipopolysaccharide (LPS)-induced injury in *Caenorhabditis elegans*.

**Methods:**

*C. elegans* individuals were stimulated for 24 h with LPS (100 μg/mL), with or without carnosine (0.1, 1, 10 mM). The survival rates and behaviors were determined. The activities of superoxide dismutase (SOD), glutathione reductase (GR), and catalase (CAT) and levels of malondialdehyde (MDA) and glutathione (GSH) were determined using the respective kits. Reverse transcription polymerase chain reaction (RT-PCR) was performed to validate the differential expression of *sod-1*, *sod-2*, *sod-3*, *daf-16*, *ced-3*, *ced-9*, *sek-1*, and *pmk-1*. Western blotting was used to determine the levels of SEK1, p38 mitogen-activated protein kinase (MAPK), cleaved caspase3, and Bcl-2. *C. elegans sek-1* (km2) mutants and *pmk-1* (km25) mutants were used to elucidate the role of the p38 MAPK signaling pathway.

**Results:**

Carnosine improved the survival of LPS-treated *C. elegans* and rescued behavioral phenotypes. It also restrained oxidative stress by decreasing MDA levels and increasing SOD, GR, CAT, and GSH levels. RT-PCR results showed that carnosine treatment of wild-type *C. elegans* up-regulated the mRNA expression of the antioxidant-related genes *sod-1*, *sod-2*, *sod-3*, and *daf-16*. The expression of the anti-apoptosis-related gene *ced-9* and apoptosis-related gene *ced-3* was reversed by carnosine. In addition, carnosine treatment significantly decreased cleaved caspase3 levels and increased Bcl-2 levels in LPS-treated *C. elegans*. Apoptosis in the loss-of-function strains of the p38 MAPK signaling pathway was suppressed under LPS stress; however, the apoptotic effects of LPS were blocked in the *sek-1* and *pmk-1* mutants. The expression levels of *sek-1* and *pmk-1* mRNAs were up-regulated by LPS and reversed by carnosine. Finally, the expression of p-p38MAPK and SEK1 was significantly increased by LPS, which was reversed by carnosine.

**Conclusion:**

Carnosine treatment protected against LPS injury by decreasing oxidative stress and inhibiting apoptosis through the p38 MAPK pathway.

## Background

Lipopolysaccharide (LPS) is a pathogen-associated molecular pattern of gram-negative bacteria that is essential for its pathogenicity [1]. LPS plays an important role in the pathogenesis of sepsis, which is a systemic inflammatory response syndrome triggered by infections of bacteria, viruses, or parasites or by toxic products and has become a major cause of mortality in intensive care units. The characteristic features of sepsis include amplification of the initial inflammatory response followed by immunosuppression and multiple organ dysfunction or failure leading to death [2, 3]. Studies have demonstrated that LPS is a main mediator of sepsis. LPS exposure may lead to an imbalanced immune response by eliciting the release of inflammatory mediators. In addition, LPS may lead to deregulated immune responses, triggering sepsis and consequently resulting in multiple organ failure [4–6].

Invertebrate models developed over the past decade have reduced the cost and complexity of mammalian assays and avoided the need for study review by institutional animal care and use committees [7]. *Caenorhabditis elegans* is a nematode used as a successful invertebrate model of pathogenesis and one of the most commonly used standard laboratory models for bacterial pathogenesis [8]. *C. elegans* has a simple lifecycle and short generation time and requires inexpensive and simple growth conditions [9]. Moreover, as nematodes have a complex innate immune system involving various signaling pathways and antimicrobial proteins and peptides, a wide range of mutant strains of *C. elegans* provides the opportunity to explore the molecular genetic determinants of pathogen toxicity [10]. *C. elegans* do not have an obvious acquired immune response, homologs of vertebrate cytokines and circulating immune cells. However, *C. elegans* possess many innate immune components that are evolutionarily conserved with vertebrates, including a single toll-like receptor and p38 mitogen-activated protein kinase (MAPK), mediating responses to infection [11]. LPS modulated the expression of selected host immune and aging-related genes in *C. elegans* [1, 12]*.* In the present study, *C. elegans* with LPS-induced injury were used to conserve the mechanisms of innate immunity and stress response.

**Fig. 1:**
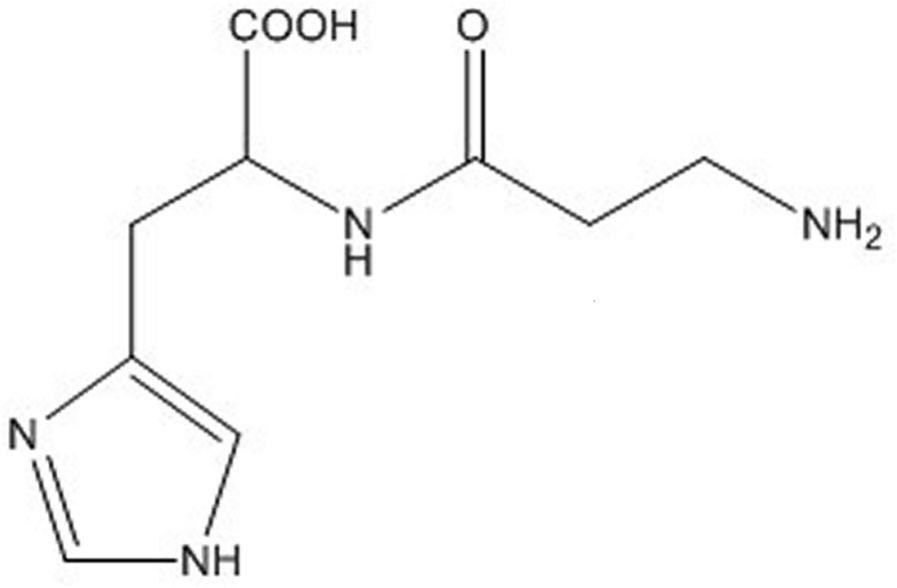
The chemical structure of carnosine

Carnosine (β-alanyl-L-histidine; Fig. 1) is a natural dipeptide that is usually abundant in excitable tissues such as nerves and muscles. Beyond its association with specific diseases, carnosine has been assigned many putative actions, such as free radical scavenging, anti-inflammation, and mobile organic pH buffering. Carnosine has in part been studied to determine its effect on oxidative stress conditions in vivo and in vitro [13–16]. Furthermore, some studies suggest that carnosine has a wholesome effect on the reduction of apoptosis by inhibiting caspase 3 increase and recovering Bcl-2 level [17]. Carnosine showed protective effects on acute lung injury in sepsis rats by enhancing the antioxidant status along with a decrease in pro-inflammatory cytokines [18]. It has been shown to have beneficial effects in reducing acute kidney injury due to septic shock in a rat model of septicemia [19]. Carnosine may be an effective treatment for oxidative damage due to liver tissue perfusion defects in cases of septic shock [20]. However, the antioxidative and anti-inflammatory effects of carnosine on *C. elegans* have not yet been determined.

The aim of the current study was to investigate the effect of carnosine on LPS-induced injury in *C. elegans*. We determined whether carnosine could effectively prevent LPS-induced injury in *C. elegans.* In addition, we elucidated the mechanism of carnosine suppression via the p38 MAPK signaling pathway and evaluated the therapeutic effect of carnosine.

## Methods

### *C. elegans* culture

*C. elegans* N2 (wild-type), *sek-1* (km2) mutants, and *pmk-1* (km25) mutants were obtained from the Caenorhabditis Genetics Center (CGC; University of Minnesota). The worms were cultured as follows: streptomycin-resistant variant strain *Escherichia coli* OP50 (OP50–1, obtained from CGC) was used as the food source for *C. elegans*. All worms were grown at 20 °C on NGM plates (1 mM CaCl_2_, 1 mM MgSO_4_, 5 g/mL cholesterol, 50 mM KH_2_PO_4_ pH 6.0, 25 mM NaCl, 1.7% agar, and 2.5 mg/mL peptone) with fresh OP50–1 as a food source.

### *C. elegans* experimental design

L4 *C. elegans* larvae were maintained on NGM agar plates containing OP50–1 at 20 °C. Treatment of *C. elegans* with carnosine or M9 buffer (5 g/L NaCl, 3 g/L KH_2_PO_4_, 6 g/L Na_2_HPO_4_, 0.25 g/L MgSO_4_^.^7H_2_O, vehicle) was performed in liquid NGM without OP50. *C. elegans* individuals (N2, km25, and km2) were exposed to M9 buffer or carnosine (0.1, 1, or 10 mM) 30 min before LPS (serotype 055:B5; Sigma, Deisenhofen, Germany) treatment. The worms were then exposed to M9 buffer or LPS (100 μg/mL) for 24 h, while carnosine was left in the buffer as a co-treatment with LPS. After 24 h, *C. elegans* individuals were washed with M9 buffer and transferred to NGM agar plates, and this step was repeated twice. They were then allowed to acclimate for 30 min before performing survival assays. Data were obtained from five independent experiments.

### Behavioral assays

Omega turn (a maneuver during which the body of the worm briefly resembles the Greek letter) and reversal tests were used to evaluate the behavior of *C. elegans*. Behavioral assays were performed on foodless NGM assay plates (60-mm diameter) [21]. Observation began 1 min after transfer to a new plate. The *C. elegans* individuals could be observed as they moved across the plate. The omega turn was defined as a sharp with a turning angle was greater than 135°with three or more body bends. Reversals are a general and important feature of the worm’s behavioral repertoire, ranging from aging and chemotaxis to escape from noxious stimuli, defined as a turn in which the head is in contact with the tail. Each animal was subjected to a maximum of five touches, and the first omega turn was scored under the observation of a stereomicroscope. A reversal was scored as any backward movement of the entire body was scored as a reversal. A SZX16 stereomicroscope (Olympus, Tokyo) was used for direct observation of behavior.

### RNA extraction and reverse transcription polymerase chain reaction

The worms were collected after exposure to M9 buffer or LPS for 24 h. Reverse transcription polymerase chain reaction (RT-PCR) was performed to verify the differential expression of *sod-1*, *sod-2*, *sod-3*, *daf-16*, *ced-3*, *ced-9*, *sek-1*, and *pmk-1*. According to the manufacturer’s instructions, total RNA was extracted using Trizol. In addition, according to the manufacturer’s protocol, 5 mg RNA was used to synthesize the first strand of complementary DNA (cDNA) using SuperScript II RNase H-Reverse Transcriptase (Invitrogen, Carlsbad, CA, USA) and used as template in subsequent PCR reactions with thermal cycling performed using an Eppendorf Mastercycler (Eppendorf, Germany). A Nano Drop 2000 spectrophotometer (Thermo, USA) was used to determine the concentration of total RNA, and RNA purity was assessed by OD260/OD280 ratios. The complementary DNA (cDNA) was amplified by using RT-PCR. Primer design is a common operation and primer amplification template is known, as following primer pairs: sod-1: (F) 5′-TGTCGAACCGTGCTGTCGCT-3′ (R) 5′-TGGACCGGCAGAAATGCATCCG-3′; *sod-2*: (F) 5′-ACCATCGGCGGAGTTGCTCA-3′, (R) 5′-AGCGTGCTCCCAGACGTCAA-3′; *sod-3*: (F) 5′-GTGGTGGACACATCAATC-3′, (R) 5′-AAGTGGGACCATTCCTTC-3′; *daf-16*: (F) 5′-GGAAGAACTCGATCCGTCACA-3′, (R) 5′-GATTCCTTCCTGGCTTTGCA-3′; *sek-1*: (F) 5′-TGCTCAACGAGCTAGACG-3′, (R) 5′-ATGTTCGACGGTTTCACG-3′; *pmk-1*: (F) 5′-CGACTCCACGAGAAGGAT-3′, (R) 5′-ATATGTACGACGGGCATG-3′; *ced-3*: (F) 5′-ACGGGAGATCGTGAAAGC-3′, (R) 5′-AGAGTTGGCGGATGAAGG-3′; *ced-9*: (F) 5′-AAAGGCACAGAGCCCACC-3′, (R) 5′-CGTTCCCATAACTCGCATC-3′; and β-actin: (F) 5′-CCAGGAATTGCTGATCGTATGCAGAA-3′, (R) 5′-TGGAGAGGGAAGCGAGGATAGA-3′. The cycle number was determined from a linear amplification curve to ensure that amplification was within the linear amplification range. The level of β-actin expression was used to normalize the gene expression. By analyzing dissociation curves, each reaction was verified to contain a single amplification product. All sample reactions were performed three times. The 2-ΔΔCt method, where ΔΔCt = [(Ct_target_ − Ct_actin_) Sample] – [(Ct_target_ − Ct_actin_) Control] with β-actin used as a reference gene were used to determine the relative changes in gene expression were determined using.

### Western blotting analysis

*C. elegans* worms were collected and homogenized in ice-cold lysis buffer containing 50 mM Tris-HCl, 1% NP-40, 150 mM NaCl, 2 mM EDTA, and 1 mM Na_3_VO_4_ (pH 7.4) using a homogenizer (Bertin Precellys 24 Technologies) after exposure to M9 buffer or LPS for 24 h. Protein samples were separated on 12% sodium dodecyl sulfate (SDS)–polyacrylamide gels and electro-transferred onto nitrocellulose membranes. The membranes were blocked by 5% fat-free milk, then incubated with primary antibodies against SEK1 (1:1000; Cell Signaling Technology, USA), p-p38 (1:1000; Abcam, UK), Bcl-2 (1:1000; Cell Signaling Technology), c-caspase3 (1:750; Cell Signaling Technology), and GAPDH (1:5000; Kangchen Bio-tech, Shanghai) for 2 h at room temperature. The membranes were washed three times with tris-buffered saline and Tween 20 buffer and incubated with IRDye 800 anti-rabbit Molecular Probe (1:8000; LI-COR Biosciences) or IRDye700 anti-mouse Molecular Probe (1:3000; LI-COR Biosciences, USA) for 2 h. Images were acquired using an Odyssey Infrared Imaging System and analyzed using Odyssey software [22].

### Evaluation of antioxidant indices

*C. elegans* worms were collected and homogenized in ice-cold lysis buffer after exposure to M9 buffer or LPS for 24 h and washed three times with M9 buffer as described above. The supernatant was centrifuged by ultrasonication. The activity of superoxide dismutase (SOD), glutathione reductase (GR), catalase (CAT) and the level of glutathione (GSH) or malondialdehyde (MDA) were detected following the instructions of T-SOD, GR, CAT, GSH or MDA assay kit (Nanjing Jiancheng Bioengineering Institute, Nanjing, China). The manipulation process was performed according to the manufacturer’s instructions.

### Statistical analysis

Statistical analyses were performed using SPSS 19.0 for Windows software. Data are presented as the mean ± standard error of the mean (SEM) and included one-way analysis of variance followed by the least significant difference or Dunnett’s T3 post-hoc test for multiple comparisons where equal variances were not assumed.

## Results

### Effect of carnosine on *C. elegans* in the presence of LPS

**Fig. 2:**
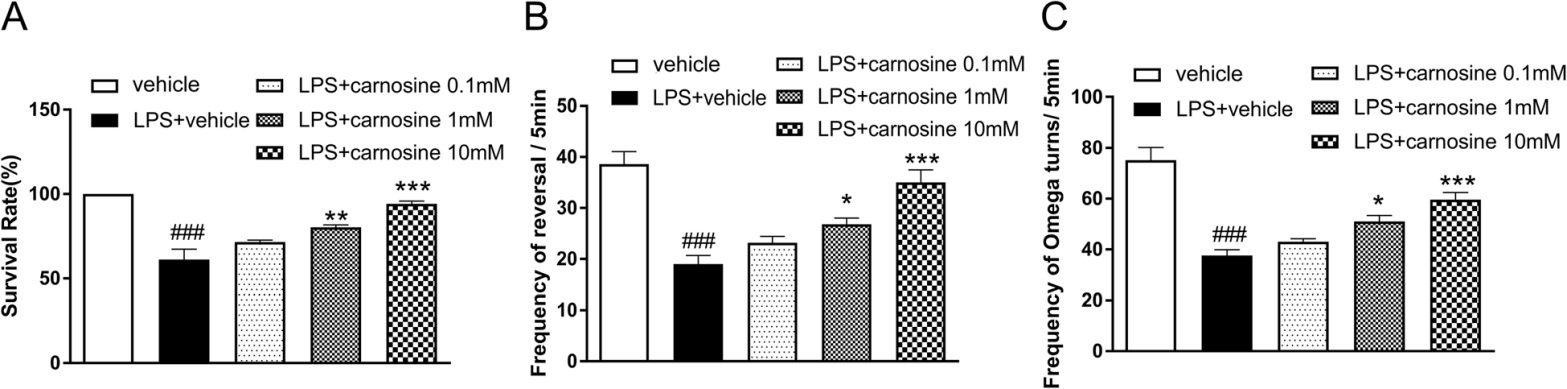
The effect of carnosine on survival (**a**), reversals (**b**) and omega turns (**c**) of *C. elegans* induced by LPS. Date are presented as means±SEM. ###*P* < 0.001, vs.the vehicle group; ***P < 0.001, **P < 0.01, *P < 0.05, vs. the LPS + vehicle group

Treatment with LPS (100 μg/mL) significantly decreased survival (61.16%) compared with that of non-treated worms (vehicle group). Carnosine significantly increased survival when treated at concentrations of 1 mM (*P* < 0.01) and 10 mM (*P* < 0.001) compared with the vehicle group. Survival was also increased in the 0.1 mM carnosine group but failed to reach a significant difference compared with that in the vehicle group (Fig. 2a). LPS exposure caused a significant reduction in the frequency of reversals (Fig. 2b) and omega turns (Fig. 2c). However, 1 and 10 mM carnosine pre-treatments significantly elevated the frequency of the behavior of *C. elegans* compared with LPS treatment. These changes were not obvious with pre-treatment with low concentrations of carnosine.

### Carnosine inhibited apoptosis induced by LPS in *C. elegans*

**Fig. 3:**
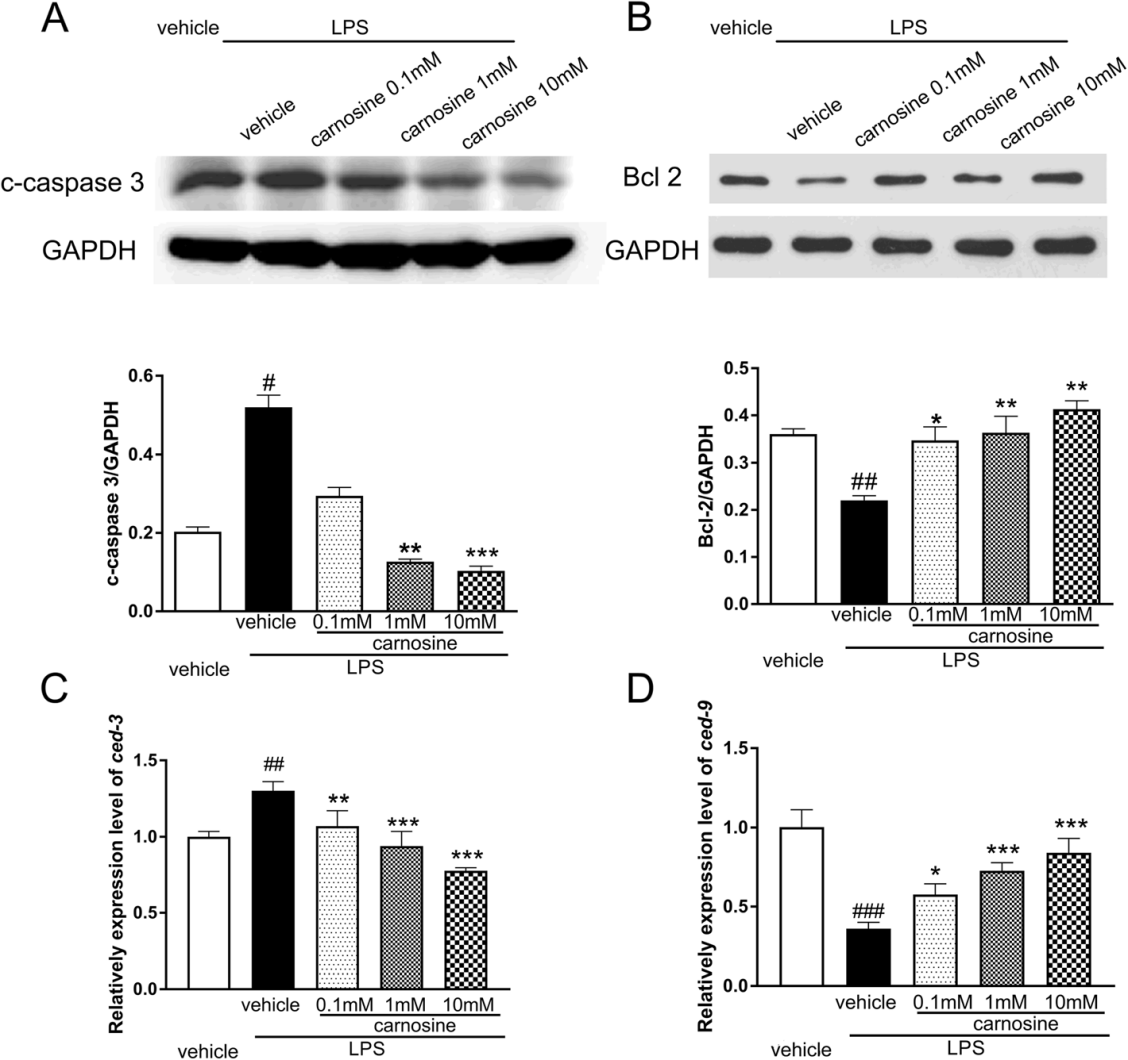
Effects of carnosine on the apoptosis. Western blot analysis of c-caspase 3 (**a**) and Bcl-2 (**b**) protein levels in *C. elegans* after LPS exposure. PCR annlysis of *ced-3* (**c**) and *ced-9* (**d**) gene expression in *C. elegans* after LPS exposure. Date are presented as means±SEM. ###*P* < 0.001, ##*P* < 0.01, #*P* < 0.05, vs. the vehicle group; ***P < 0.001, **P < 0.01, *P < 0.05, vs. the LPS + vehicle group

Western blotting results showed that the level of cleaved caspase 3 protein was significantly elevated in the LPS-treated group (*P* < 0.05), and this increased expression was down-regulated by treatment with 1 and 10 mM carnosine (Fig. 3a). In contrast, the levels of Bcl-2 expression were significantly decreased by exposure to LPS (*P* < 0.01), but treatment with carnosine effectively elevated the levels of Bcl-2 expression in LPS-treated worms (Fig. 3b). The effect of carnosine on gene expression of the apoptosis inhibitor *ced-9* (the Bcl-2 homolog) and *ced-3* (the initiator procaspase) was evaluated by using RT-PCR. As shown in Fig. 3c and d, compared with the control group, *ced-9* levels were significantly decreased and *ced-3* levels were significantly increased in the LPS group. Carnosine effectively reversed LPS-induced changes in gene expression of *ced-9* and *ced-3* (*P* < 0.001).

### Carnosine ameliorated oxidative stress induced by LPS in *C. elegans*

**Fig. 4:**
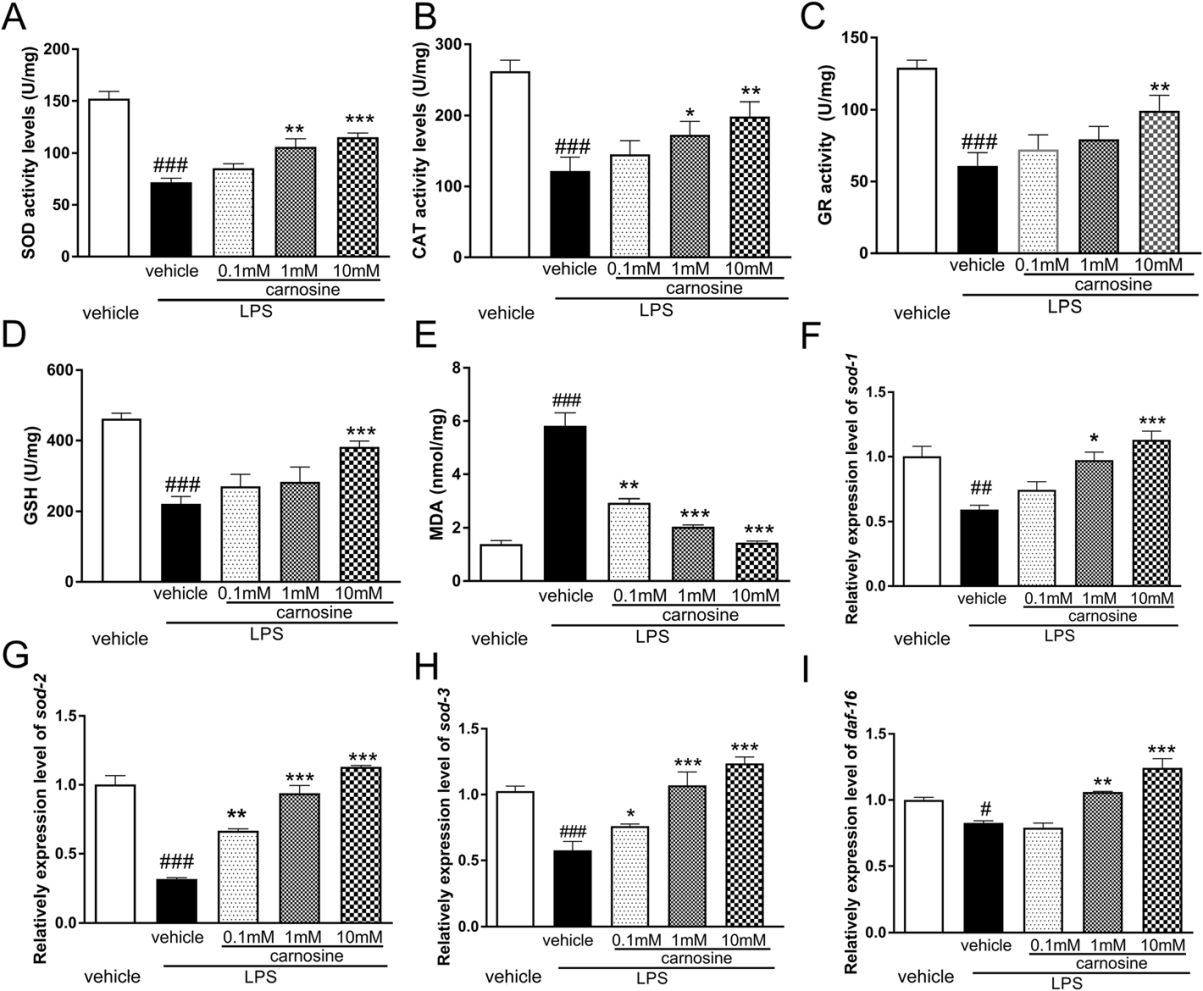
Carnosine ameliorates oxidative stress in *C. elegans* induced by LPS. **a** The levels of SOD (**a**), CAT (**b**), GSH (**c**), GR (**d**) and MDA (**e**) were detected in *C. elegans* after LPS exposure. The levels of sod-1 (**f**), sod-2 (**g**), sod-3 (**h**) and daf-16 (**i**) mRNA in *C. elegans* were decreased by LPS treatment, and were significantly reversed by carnosine. Date are presented as means±SEM. ###*P* < 0.001, vs. the vehicle group; ***P < 0.001,**P < 0.01, *P < 0.05, vs. the LPS + vehicle group

The levels of SOD, CAT, GSH, and GR were significantly decreased in LPS group compared with those in the vehicle group (*P* < 0.001). However, treatment with carnosine effectively elevated these levels in *C. elegans* treated with LPS (Fig. 4a-d). The levels of MDA in *C. elegans* were increased by LPS treatment (*P* < 0.001), but this was significantly reversed by pre-treatment with carnosine (Fig. 4e). The levels of *sod-1*, *sod-2*, *sod-3,* and *daf-16* mRNA in *C. elegans* decreased in response to LPS treatment (Fig. 4f-i), but these were significantly reversed by carnosine treatment.

### Effect of carnosine on p38 MAPK signaling pathway of *C. elegans* treated with LPS

**Fig. 5:**
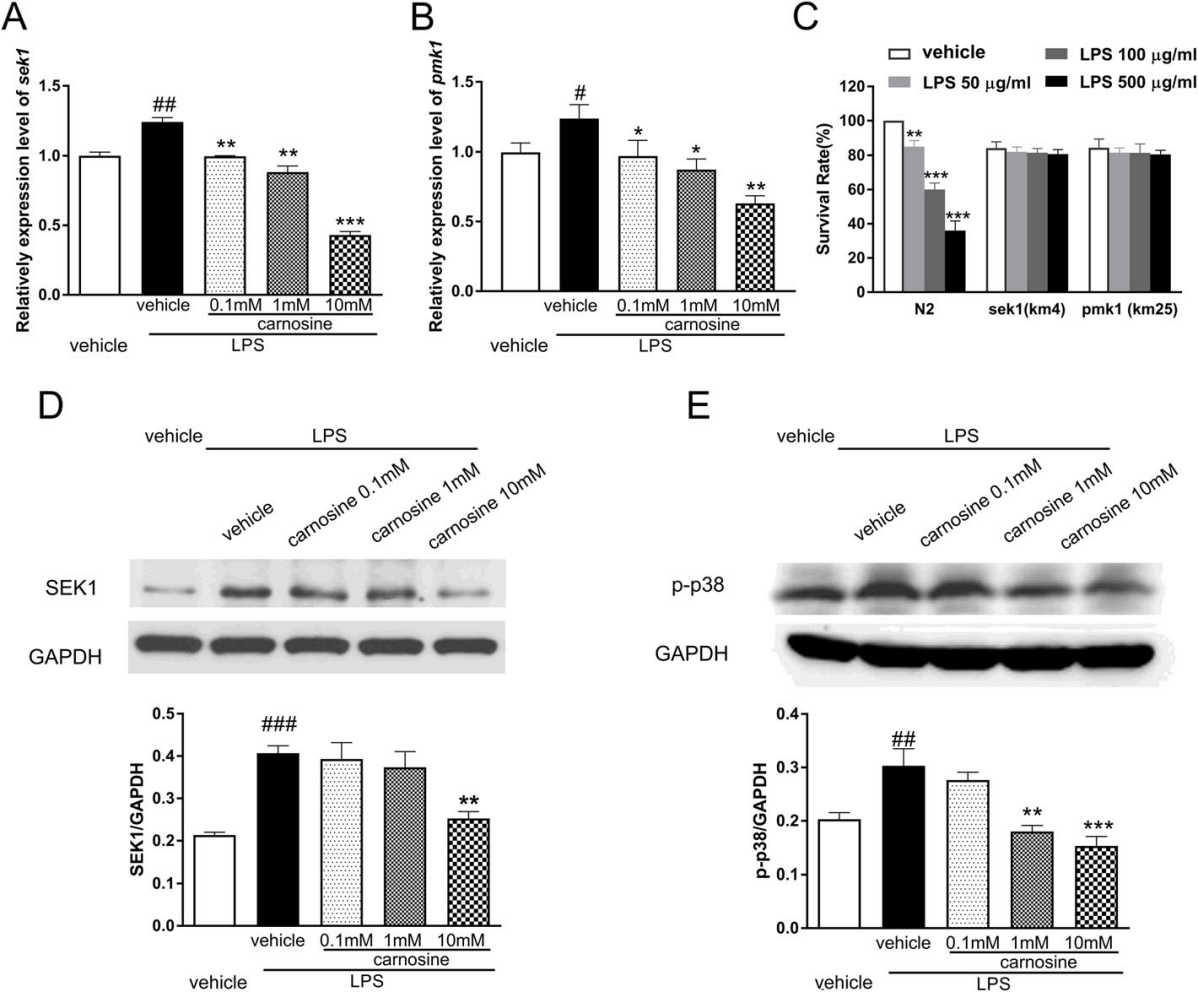
Effects of carnosine on the expression of p38 MAPK signaling pathway. The levels of sek-1 (**a**) and pmk-1 (**b**) mRNA in *C. elegans* were detected by PCR. **c** The survival rate of worms were not altered by LPS treatment in pmk-1(km25), sek-1(km4) mutants. Western blot analysis of SEK1 (**d**) and p-p38 (**e**) protein levels in *C. elegans* after LPS exposure. Date are presented as means±SEM. ###*P* < 0.001, ##*P* < 0.01, #*P* < 0.05, vs.the vehicle group; ***P < 0.001, **P < 0.01, *P < 0.05, vs. the LPS + vehicle group

After exposure to LPS for 24 h, the transcriptional expression of genes *pmk-1* and *sek-1* significantly increased compared with that in the vehicle group. However, treatment with carnosine effectively reduced *pmk-1* and *sek-1* gene expression in worms treated with LPS (Fig. 5a and b). As shown in Fig. 5c, the survival rate of worms was not altered by LPS treatment in *pmk-1* (km25) mutants or *sek-1* (km4) mutants, suggesting that *pmk-1* and *sek-1* may play pivotal roles in mediating LPS-induced death in *C. elegans*. Compared with that in the vehicle group, both phosphorylated p38 (p-p38) and SEK1 expression was greatly increased in the LPS group of *C. elegans* worms. Nonetheless, the expression of p-p38 and SEK1 was reduced following pre-treatment with carnosine (Fig. 5d and e).

## Discussion

The present study evaluated the potential of carnosine as an antioxidant agent using a *C. elegans* model. Carnosine increased the survival rates and frequency of normal behavior of worms treated with LPS group significantly. We found that carnosine significantly attenuated oxidative injury and prevented apoptosis. The protective effect of carnosine on *C. elegans* may be related to the p38 MAPK signaling pathway.

Carnosine has been reported to possess anti-inflammatory and antioxidative properties [13]. However, the effect of carnosine on LPS-induced injury in *C. elegans* has not been reported. In the current study, LPS exposure resulted in significant nematode mortality and reduced frequencies of reversals and omega turns, which were effects rescued by carnosine. As an important enzyme that catalyzes the dismutation of superoxide, SOD plays an important role in the first defense line of cell oxidative damage [23]. Using CAT, SOD can convert ROS into hydrogen peroxide, resulting in an oxidant effect [24]. GSH is an important biological free radical scavenger and antioxidant and maintained endogenous redox homeostasis [25]. GR is also an important enzyme that maintains the levels of reduced GSH in cells [26], while MDA is an end product of polyunsaturated fatty acid peroxidation, which is promoted by oxidative stress [27]. We compared the levels of important enzymes in *C. elegans* worms following treatment with LPS and carnosine. MDA levels were markedly increased and GSH levels were decreased in *C. elegans* treated with LPS, demonstrating the occurrence of oxidative stress. The activities of various enzymes regulating oxidative stress, including SOD, GR, and CAT, were significantly decreased following exposure to LPS. The administration of carnosine ameliorated LPS-induced oxidative stress, as evidenced by the significant inhibition on LPS-induced increase of MDA and decrease of SOD, GR, CAT, and GSH. The transcription factor *daf-16* is a homologous gene of mammal FOXO proteins in *C. elegans* that play critical roles in stress response [28]. *Daf-16*-knockout worms are highly sensitive to oxidative stress, and they exhibit decreased lifespan [29, 30]. The mammalian ortholog of SIRT1, the *sir-2.1* gene, is known to modulate oxidative stress responses and longevity through *daf-16* [31, 32]. The antioxidative effect of carnosine was confirmed by the reversal of downregulation of three *sod* genes and *daf-16*. The dose of carnosine (0.1–10 mM) in our research is similar to the dose in humans (0.5–2 g/day) at which carnosine exhibited protective effects on LPS-induced oxidative damage [33–35]. Caspase 3, a frequently activated death protease, catalyzes the specific cleavage of many key cellular proteins. For instance, caspase 3 is required for apoptotic scenarios in tissues and cells and is indispensable for DNA fragmentation and apoptotic chromatin condensation [36]. Meanwhile, Bcl-2, an anti-apoptotic protein, sequesters and inhibits pro-apoptotic proteins and prevents apoptosis by blocking the key steps of caspases activation [37]. Carnosine effectively elevated the expression levels of Bcl-2 and reduced the levels of caspase 3 in *C. elegans* worms treated with LPS. The *C. elegans ced-3* gene is essential for apoptosis and encodes apoptotic caspases [38]. *ced-3* is a homolog of mammalian caspase 3, which is a protease that eventually kills cells. The activation and regulation of *ced-3* during apoptosis plays important roles in the activation and function of caspases and apoptosis. In contrast to *ced-3*, *C. elegans* Bcl-2 homolog *ced-9* plays a central role in preventing cell death in worms [39]. Carnosine effectively reversed the decrease of *ced-9* and increase of *ced-3* induced by LPS. Our results from both behavioral and western blotting analyses show that LPS-induced apoptosis in *C. elegans* could be rescued by carnosine.

It is also known that p38 MAPK plays a key role in the process of sepsis caused by LPS [40]. As a member of the MAPK superfamily, p38 is activated by various cellular stresses and ligands. In *C. elegans*, pmk-1 is a p38 MAPK homolog, which is associated with the apoptotic regulation of germ cells, innate immune response, and oxidative reactions [41, 42]. SEK-1 is also an important component of the p38 pathway, which is required for innate immunity in *C. elegans* [43]. SEK-1 is a MAPK kinase and the *C. elegans* ortholog of mammalian MKK3/MKK6 in the p38 MAPK pathway [44]. Our results from both PCR and western blotting analyses demonstrated that LPS significantly increased the transcriptional expression of *pmk-1* and *sek-1* genes and the expression level of p-p38 and SEK1. We therefore investigated the effects of carnosine on the local activities of the p38 MAPK signaling pathway in LPS-induced *C. elegans*. The results showed that carnosine inhibited the expression of the p38 MAPK signaling pathway. Therefore, carnosine might act as a suppressor of the p38 MAPK signaling pathway. To confirm the function of the p38 MAPK signaling pathway in regulating LPS toxicity, we investigated LPS toxicity in worms with mutations in *pmk-1*(km25) and *sek-1*(km4), which are genes associated with the p38 MAPK signaling pathway in nematodes. Our results revealed that *pmk-1* and *sek-1* play pivotal roles in mediating LPS-induced death in *C. elegans*, which further confirmed that carnosine protected the nematodes from LPS-induced damage through the p38 MAPK signaling pathway.

## Conclusions

In conclusion, our study reveals a potential protective role of carnosine in LPS-induced *C. elegans* model. Carnosine treatment protected against LPS-induced injury by decreasing oxidative stress and inhibiting apoptosis through the p38 MAPK pathway. Our findings provide insights into the impact of carnosine on conserved mechanisms of innate immunity and stress response.

## Supplementary Information

**Additional file 1.**

## Data Availability

All data generated and analyzed during this study are included in this article. The datasets used and/or analyzed during the current study are available from the corresponding author on reasonable request.

## Abbreviations

CAT: Catalase
cDNA: complementary DNA
*C. elegans*: *Caenorhabditis elegans*
ELISA: Enzyme-linked Immunosorbent assay
GR: Glutathione reductase
GSH: Glutathione
LPS: Lipopolysaccharide
MAPK: Mitogen-activated protein kinase
MDA: Malondialdehyde
ROS: Reactive oxygen species
RT-PCR: Reverse transcription polymerase chain reaction
SDS: Sodium dodecyl sulfate
SOD: Superoxide dismutase
TNF-α: Tumor necrosis factor alpha
