# Supplementary figures and images for: Lipopolysaccharide exposure induces oxidative damage in Caenorhabditis elegans: protective effects of carnosine
Source: BMC Pharmacol Toxicol. 2020 Dec 3;21:85. doi: 10.1186/s40360-020-00455-w (PMC7713333; doi:10.1186/s40360-020-00455-w)

Figure 3A：


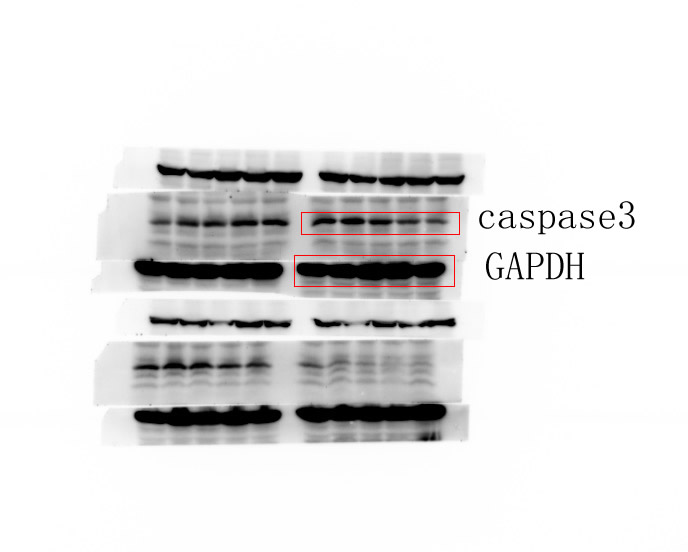


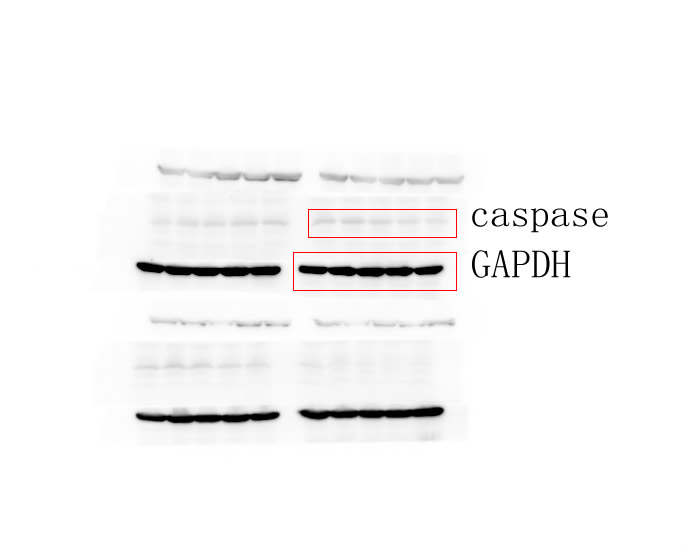


Figure 3B：


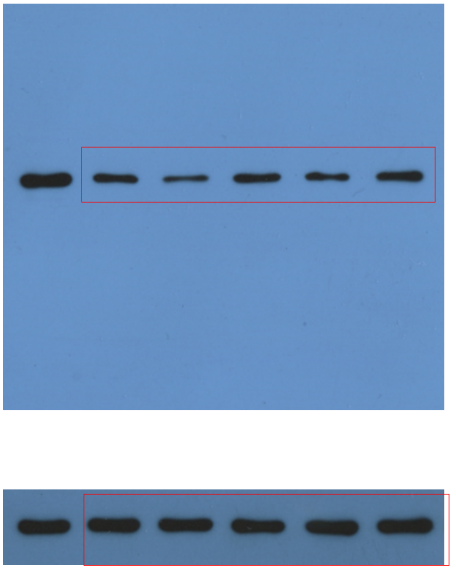


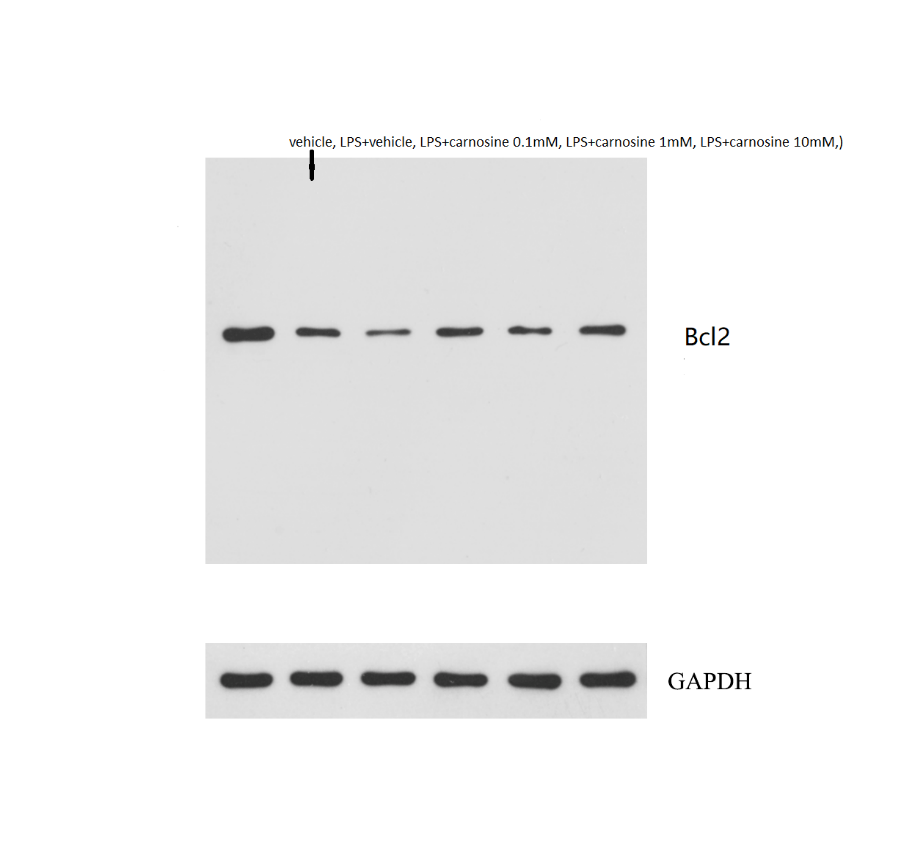


Figure 5D:


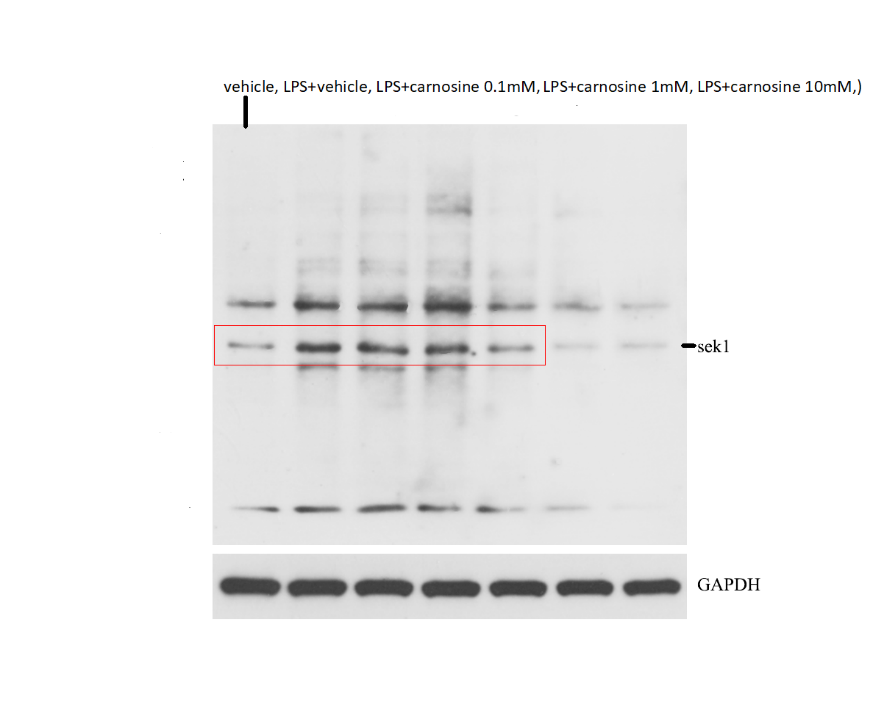


Figure 5E :


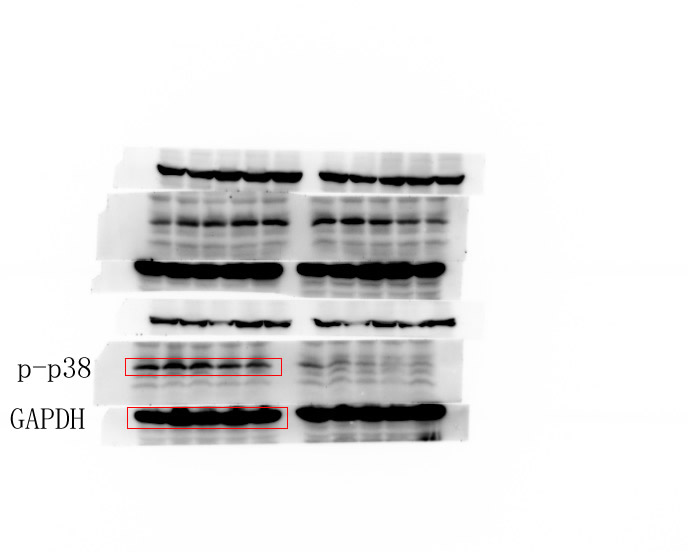


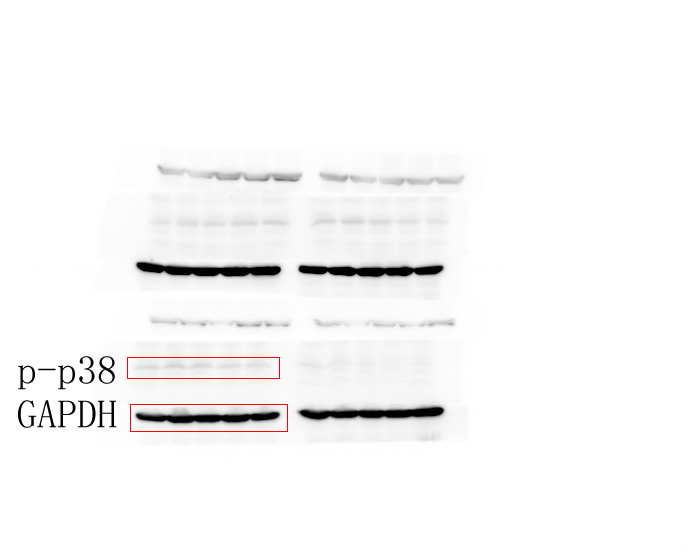

Supplement: Supplementary file 1 — Additional file 1. [file 40360_2020_455_MOESM1_ESM.docx]
